# Effects of Per- and Polyfluoroalkyl Compounds (PFASs) on Anaerobic Granular Sludge: Methane Production and Microbial Community Composition

**DOI:** 10.1007/s00248-026-02793-4

**Published:** 2026-07-09

**Authors:** Davide Righetti, Borja K. Soliman Tamayo, Silvia Lampis, Piet N. L. Lens

**Affiliations:** 1 Department of Biotechnology, University of Verona, Strada le Grazie, 15, 37134 Verona, Italy; 2 Department of Biotechnology, VUCC-DBT Verona University Culture Collection, University of Verona, Strada le Grazie, 15, 37134 Verona, Italy; 3 University of Galway, University Road, H91 TK33, Galway, Ireland

**Keywords:** Anaerobic granular sludge, Biomethane, Per- and polyfluoroalkyl substances, Toxicity, Microbiome

## Abstract

PFASs are a group of pollutants ubiquitous in the environment, for which their effects on the microbial community composition and activity of anaerobic granular sludge are still poorly understood. With our study, we aimed to provide insight into the impact of four PFASs on the methane yield of anaerobic granular sludge (AGS) using acetate as the substrate. Anaerobic granular sludge was exposed to different concentrations of 1H,1H,2H,2H-Tridecafluorooctan-1-ol (6:2FTOH), 1H,1H,2H,2H-Perfluoro-1-decanol (8:2FTOH), Tridecafluorohexane-1-sulfonic acid (PFHxS) and Perfluorooctanoic acid (PFOA). While the two fluorotelomers caused marginal changes in methane production, PFHxS and PFOA greatly inhibited acetoclastic methanogenesis. Depending on the concentration, the AGS could recover its activity after a certain number of hours when incubated with PFASs. The 50% Inhibitory Concentration (IC_50_) of the methane production was estimated to be 278.98 (± 3.2) mg/L for PFOA and 1,091.9 (± 26.78) mg/L for PFHxS. PFASs exposure also influenced the archaeal and eubacterial communities. The most significant change was observed with the eubacterial community, which showed an increase in the relative abundance of the genus *Sulfurospirillum* in the samples treated with 2,000 mg/L of 8:2FTOH and 600 mg/L of PFOA, whose presence grew to represent 16.65% and 45.4% of all reads in those samples. These findings provide insight into the differential impact of PFASs on methanogenic processes and highlight their potential to disrupt key microbial functions in anaerobic systems.

**Supplementary Information:**

The online version contains supplementary material available at https://doi.org/10.1007/s00248-026-02793-4.

## Introduction

Per- and Poly- Fluoroalkyl Substances (PFASs) are a class of highly fluorinated alkyl compounds characterized by remarkable chemical and thermal stability, as well as amphiphilic properties. Due to their persistance and variety of applications, from consumer products to industrial uses, they have become a widespread environmental concern [1, 2].

PFASs emissions are largely imputable to industrial effluents and landfill leachates [3, 4]. Concentrations of perfluoroalkyl acids (PFAAs) in wastewaters vary but can reach 1,000 ng/L [5], with 563–638 Kg of PFASs reaching wastewater treatment plants (WWTPs) as estimated for the U.S. in 2013 [6]. Landfill leachate is a major contributor, where PFASs concentrations may reach up to 93,100 ng/L prior to dilution in wastewaters.

Among the PFASs, polyfluorinated compounds, like fluorotelomer alcohols (FTOHs) [7, 8] and fully perfluorinated compounds, including perfluoroocatanoic acid (PFOA) and perfluorohexane sulfonate (PFHxS) are widely detected in the environment [9, 10]. The effects of PFASs on anaerobic treatment systems have begun to receive attention. In particular, PFOA exposure in anaerobic granular sludge (AGS) has been shown to affect the chemical oxygen demand (COD) removal efficiency, alter microbial community composition and modify the protein content of extracellular polymeric substances (EPS) [11, 12]. However, its impact on methane production remains uncertain with only a few studies investigating this process. Concentrations of PFOA under 10 mg/L seem to increase methane yields [13], while higher concentrations, up to 100 mg/L, can induce the opposite effect [11]. While the effects of PFOA have been partially investigated, the impact of PFHxS and FTOH on AGS remains largely unexplored.

Anaerobic granular sludge (AGS) consists of dense and structured microbial aggregates that enable efficient wastewater treatment under anaerobic conditions [14]. These systems rely on complex consortia of bacteria and archaea, where bacteria contribute to the degradation of organic matter and structural integrity of the granules, while methanogenic archaea are responsible for methane production [12, 15]. Acetoclastic methanogenesis represents a key pathway in anaerobic carbon mineralization, being responsible for a substantial fraction (up to ~ 50–70%) of methane production in both natural and engineered systems [16, 17]. This process plays a central role in maintaining the stability of anaerobic microbial consortia by removing acetate, a major intermediate of organic matter fermentation [18, 19]. Despite increasing attention to PFASs contamination, only a limited number of studies have addressed their effects on methanogenic processes in AGS. This knowledge gap is particularly relevant given the ecological importance of methanogenesis.

Inhibition of acetoclastic methanogenesis may therefore lead to the accumulation of volatile fatty acids, particularly acetate, resulting in process imbalance, acidification, and disruption of syntrophic interactions within the microbial community [20, 21]. Such effects can propagate across the entire anaerobic food web, ultimately impairing carbon fluxes and methane production. From a broader ecological perspective, this pathway constitutes a major route for the conversion of organic carbon into methane in anoxic environments such as sediments, wetlands, and anaerobic digesters [22].

In this context, the present study evaluates the effects of different concentrations of four PFASs (6:2FTOH, 8:2FTOH, PFHxS and PFOA) on the acetoclastic methanogenesis in AGS. Methane production was monitored to determine the 50% inhibitory threshold (IC_50_) while changes in the microbial community composition were assessed through metabarcoding analysis. Additionally, the structural integrity of the granules was examinated using transmission electron microscopy. The aim of this study was to assess the impact of PFAS on methanogenic acetoclastic activity, rather than their fate in anaerobic environments.

## Materials and Methods

### Source of Biomass

The AGS used was provided by Carbery (Ballineen, Co Cork, Ireland) and used in their bioreactors to treat dairy wastewater.

### Chemical Compounds

Four PFASs were used in this study: the two fluorotelomers alcohols 1H,1H,2H,2H-Tridecafluorooctan-1-ol or 6:2FTOH (Neo Biotech, Nanterre, France) and 1H,1H,2H,2H-Perfluoro-1-decanol or 8:2FTOH (Merk Life Science S.r.l., Milan, Italy); and two perfluorinated compounds: Tridecafluorohexane-1-sulfonic acid or PFHxS (Merk Life Science S.r.l., Milan, Italy) and perfluorooctanoic acid or PFOA (Merk Life Science S.r.l., Milan, Italy). Stock solutions were prepared in dimethyl sulfoxide (DMSO; Thermo Fisher Scientific, Waltham, USA) at a concentration of 200 g/L.

The acetate stock solution (3 M) was prepared in deionized water, starting from sodium acetate anhydrous (Thermo Fisher Scientific, Waltham, USA).

### Specific Methanogenic Assay (SMA)

#### Buffer

The buffer comprised of 0.56 g/L of L-cysteine hydrochloride anhydrous (Thermo Fisher Scientific, Waltham, USA), 0.5 mg/L of resazurin sodium salt (SigmaAldrich, Arklow, Ireland), 20 mM HEPES (Thermo Fisher Scientific, Waltham, USA) and 12.72 g/L NaCl (SigmaAldrich, Arklow, Ireland), diluted in deionized water to obtain a violet solution. It was then boiled until it became transparent.

#### Operational Conditions

Tests were performed in duplicates as described by Lawrence et al. (2025) in sealed 120 mL glass bottles with SMA buffer for a final working volume of 50 mL. AGS at 3.7 gVS/L (grams of Volatile Solids per Liter) was acclimated in the buffer for three days under nitrogen gas. The anaerobic atmosphere was created through an Anaerobe Gas-Exchange System (G.R. Instruments B.V., Wijk bij Duurstede, Netherlands). On the third day, acetate (final concentration 30 mM) and DMSO (for the positive controls) or PFAS were added, and the bottle’s headspace atmosphere was substituted with nitrogen gas. Different concentrations of PFAS were used, starting from 100 mg/L and then adjusted to assess their inhibitory effects: 6:2FTOH and 8:2FTOH (100–600–2,000 mg/L), PFHxS (100–600–900–1,200–1,500–2,000 mg/L) and PFOA (100–200–250–300–350–400–600 mg/L). Intermediate concentrations of PFHxS and PFOA, i.e. between 900 and 1,500 mg/L of PFHxS and between 200 and 400 mg/L of PFOA were tested as biological triplicates to strengthen their statistical significance. For the positive controls, DMSO was added as an equal volume than the PFAS solution, testing all concentrations. Negative controls, i.e. the AGS in the buffer without additions, were also tested. Tests were performed for five days after the addition of acetate and DMSO/PFAS. Twice a day, the pressure (Bar) inside the headspace of the bottle was measured, before and after the collection of a gas sample [23].

Methane values were expressed as mg/Kg of VS of sludge. The values were first converted to standard temperature and pressure (STP: 273.15°K and 1,013.25 mBar) and adjusted for the presence of water vapour. The values were then elaborated as per Wu et al. (2011) with the following variation: the values were normalized on the control and expressed as a percentage, with the 100% value being the one obtained from the positive control and the 0% value the one produced by the negative control [24].

### Analytics

pH measurements of the media were taken at the start and at the end of each set of experiments. This parameter was monitored since it highlights acetate consumption and methane production [25]. The gas phase of the incubations was analyzed with a Gas Chromatograph – Mass Spectrometry (GC-MS) 7890B CG system (Agilent Technologies, Santa Clara, USA) as per Lawrence et al. (2025).

The 50% Inhibitory Concentration (IC_50_) was calculated on the methane yield expressed as percentage, excluding the first 24 h for consistency, as described by Wu et al. (2011). Data were analyzed plotting % of methane produced by time in excell.

### Transmission Electron Microscopy (TEM) Observations of AGS Granules

Collected samples were washed twice with deionized water, fixed for two hours in fixative solution (2% glutaraldehyde in 0.1 M cacodylate buffer pH 7.2) and stored in cacodylate (0.2 M) buffer. Then stained using a secondary fixative solution (1% osmium tetroxide in 0.1 M sodium cacodylate buffer pH 7.2) for two hours and dehydrated through a graded series of ethanol. After the dehydration, single granules were embedded in epoxy resin and sliced into ultrathin sections of 80 nm. Observations were done with a Hitachi 7500 TEM microscope (Hitachi High-Tech, Tokyo, Japan) [26].

### Microbial Community Characterization

DNA was extracted from 2 mL of AGS granules with the DNeasy PowerSoil Pro kit^®^ (Qiagen; Hilden, Germany) following the manual’s instructions. Two biological replicates for each chosen condition were used. Samples were: T0 (the original AGS, previous to any tests), CTRL+ (AGS fed with acetate), C62 (AGS subjected to acetate and 2000 mg/L of 6:2FTOH), C82 (AGS subjected to acetate and 2000 mg/L of 8:2FTOH), CPx (AGS subjected to acetate and 2000 mg/L of PFHxS) and CPo (AGS subjected to acetate and 600 mg/L of PFOA).

For DGGE-PCR analyses, the DNA was amplified through a nested PCR following two steps of amplification for Eubacteria and a semi-nested PCR for Archaea. In the first step, eubacterial 16S rRNA was amplified with primers fD1 (5’- AGAGTTTGATCCTGGCTCAG) and rP2 (5’- ACGGCTACCTTGTTAGCACTT) [27]. PCR consisted of an initial denaturation at 94 °C for 4 min, then 30 cycles of denaturation at 94 °C for 1 min, annealing at 42 °C for 1 min and elongation at 72 °C for 2 min, final extension was set at 72 °C for 5 min. The second PCR to amplify the V3 eubacterial region, the two PCRs for the archaeal DNA, and the settings of the DGGE parameters were done according to Vargas et al. (2013) [28].

Dendrograms for DGGE were created with the UviBandMap software (UVITEC, Cambridge, UK).

### Microbiome Analyses of Anaerobic Granular Sludge

Single DNA extracts per conditions were sent to Eurofins Genomics (Ebersberg, Germany) for microbiome analysis following the provider’s standard protocols. Amplicon sequencing targeting the V3–V4 hypervariable region of the 16 S rRNA gene was performed for both Bacteria and Archaea. Prior to sequencing, DNA quality and quantity were assessed using a Qubit 4 Fluorometer (Thermo Fisher Scientific, Waltham, USA). Raw sequencing data were processed by Eurofins Genomics using their internal standardized bioinformatics pipeline, which included demultiplexing, primer removal, read merging, quality filtering, and taxonomic assignment for microbial community profiling. Raw data have been submitted to the National Center for Biotechnology Information (NCBI) under the BioProject PRJNA1416243. The resulting taxonomic profiles were used to generate relative abundance plots and to calculate alpha and beta diversity metrics. Further details on the bioinformatics workflow are available from the service provider upon request.

Microbial community dissimilarities were assessed with R (version 4.6.0) using Principal Coordinates Analysis (PCoA) based on a sample-to-sample distance matrix and Principal Component Analysis (PCA) based on sample-to-sample microbial community variation via Brey-Curtis. Visualization of ordination results was carried out using two-dimensional plots of the first principal coordinates [29, 30]. Due to the absence of biological replicates for each treatment, the analysis was conducted in a descriptive framework.

## Results

### Effects of PFASs on Methanogenic Activity

#### Shift in Methane Productivity

**Fig. 1:**
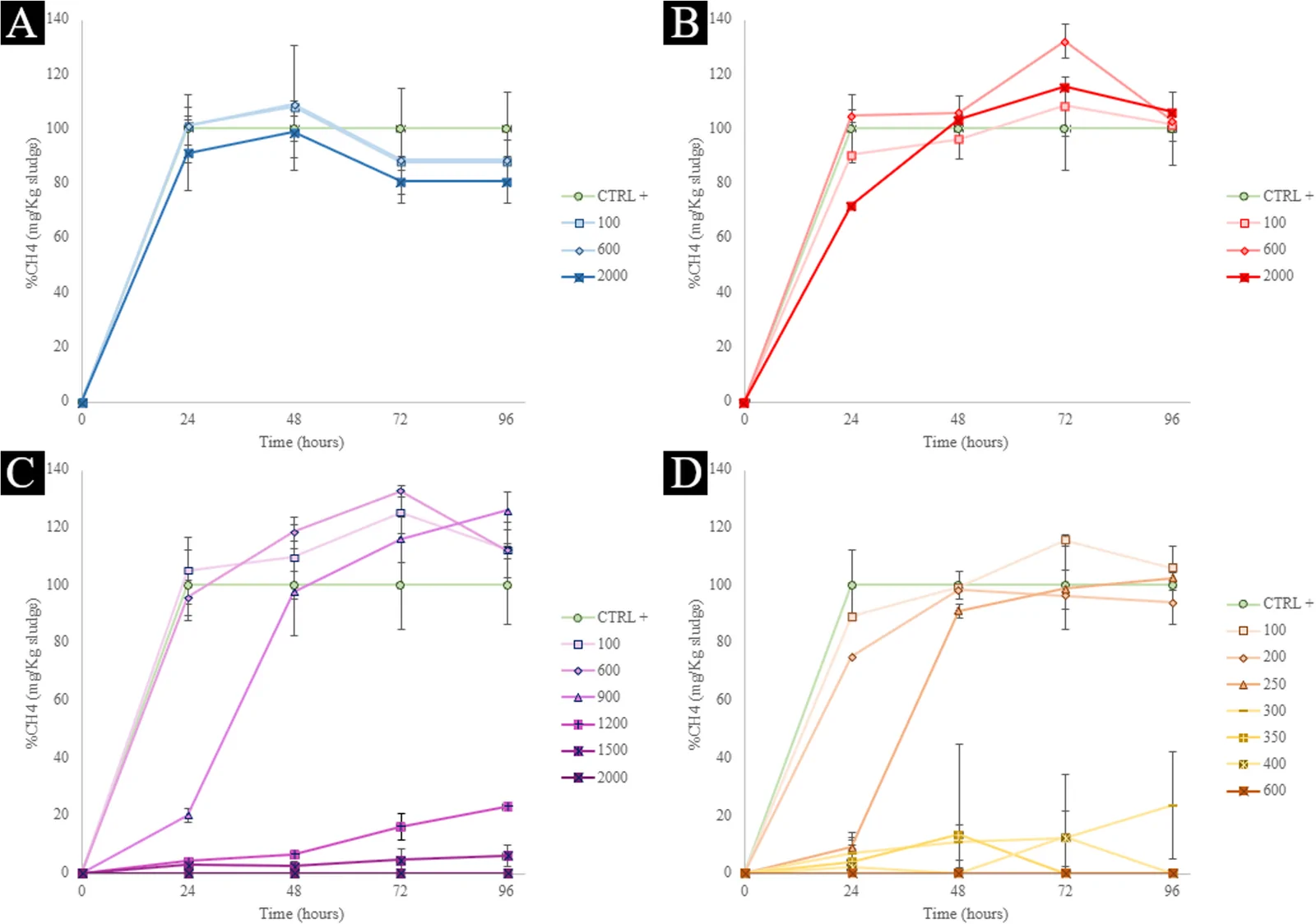
Methane yields, normalized on the control conditions, collected from tests conducted with the four PFAS compounds investigated. 0 and 100% are respectively the amount of methane observed under negative and positive control conditions (CTRL+), i.e. without or with acetate as the substrate. Pictures report the results of the methane produced when the AGS was exposed to different concentrations of (**A**) 6:2 FTOH; (**B**) 8:2 FTOH; (**C**) PFHxS and (**D**) PFOA

Under positive control conditions (CTRL+) methane production was regular and, on average, reached 10,739 (± 463.52) mL/Kg of sludge. The negative control had a minimal endogenous methane production of 178.98 (± 259.19) mL/Kg. When PFASs were added, methane yields shifted in response to the concentration and type used (Fig. 1); the applied PFAS concentration range (100–2,000 mg/L) was tested to evaluate their toxicity via IC_50_ determination (Fig. S1). All PFASs were tested at an initial concentration of 100 mg/L. This concentration seemed to affect only marginally the acetoclastic methanogenic activity of the AGS, and had to be increased in all subsequent experiments. 100 mg/L caused an initial lag in methane yields with 8:2FTOH (Fig. 1B) and PFOA (Fig. 1D), recovered by the 48th hour, and an increase in methane yields with PFHxS, up to 25% (Fig. 1C). 600 mg/L instead had a more marked effect, with PFOA totally inhibiting methane production, while it seemed to provoke a late increase in methane accumulation with 8:2FTOH and PFHxS. The highest concentration, i.e. 2,000 mg/L, decreased methane yield (25%) by 72nd hour with 6:2FTOH (Fig. 1A), while it caused a slight increase with 8:2FTOH and total inhibition with PFHxS.

PFHxS and PFOA had a marked dose-dependent effect. PFHxS decreased methane yields in the first 48 h in all treatments above 100 mg/L. At 900 mg/L, the initial production decreased to 95.5% and 20.1%, respectively, rising and reaching a maximum at 72 h, with an increase of 32.6% and 15.8%. Exposing the AGS to higher concentrations of PFHxS had only negative effects, and only samples exposed to 1,200 mg/L of PFHxS were able to partially recover the methane production, but never above the 23.1% of the control (Fig. 1C).

PFOA had a more dire effect than PFHxS. 100 mg/L was the only concentration that didn’t cause a drastic decrease in methane yield. With 200 and 250 mg/L, the AGS required 48 to 72 h to reach methane production levels equal to the control. 300 mg/l were sufficient to permanently and drastically reduce acetoclastic methane production: this incubation reached only 23.9% of the control after 96 h. From 350 mg/L onward, methane production results halted and were highly erratic (Fig. 1D).

#### Evaluation of the IC_50_ Parameter

The IC_50_ of methane production using acetate as the substrate was calculated only for PFHxS and PFOA. It amounted to 278.98 (± 3.2) mg/L for PFOA and 1,091.9 (± 26.8) mg/L for PFHxS (Fig. S1).

#### Evaluation of pH Changes

pH was measured at the start (T0) and the end (Tf) of the test period (Table S1). It was 7.28 (± 0.05) at T0 and later shifted to 7.6 (± 0.08) in the positive control conditions, i.e. with acetate. Negative controls, without acetate, showed a minor increase in pH to 7.34 (± 0.07).

FTOHs had a minimal influence on the pH shift, while no pH shift occurred with PFHxS and PFOA at concentrations higher than, respectively, 1,200 mg/L and 300 mg/L (Table S1).

### TEM Observations

**Fig. 2:**
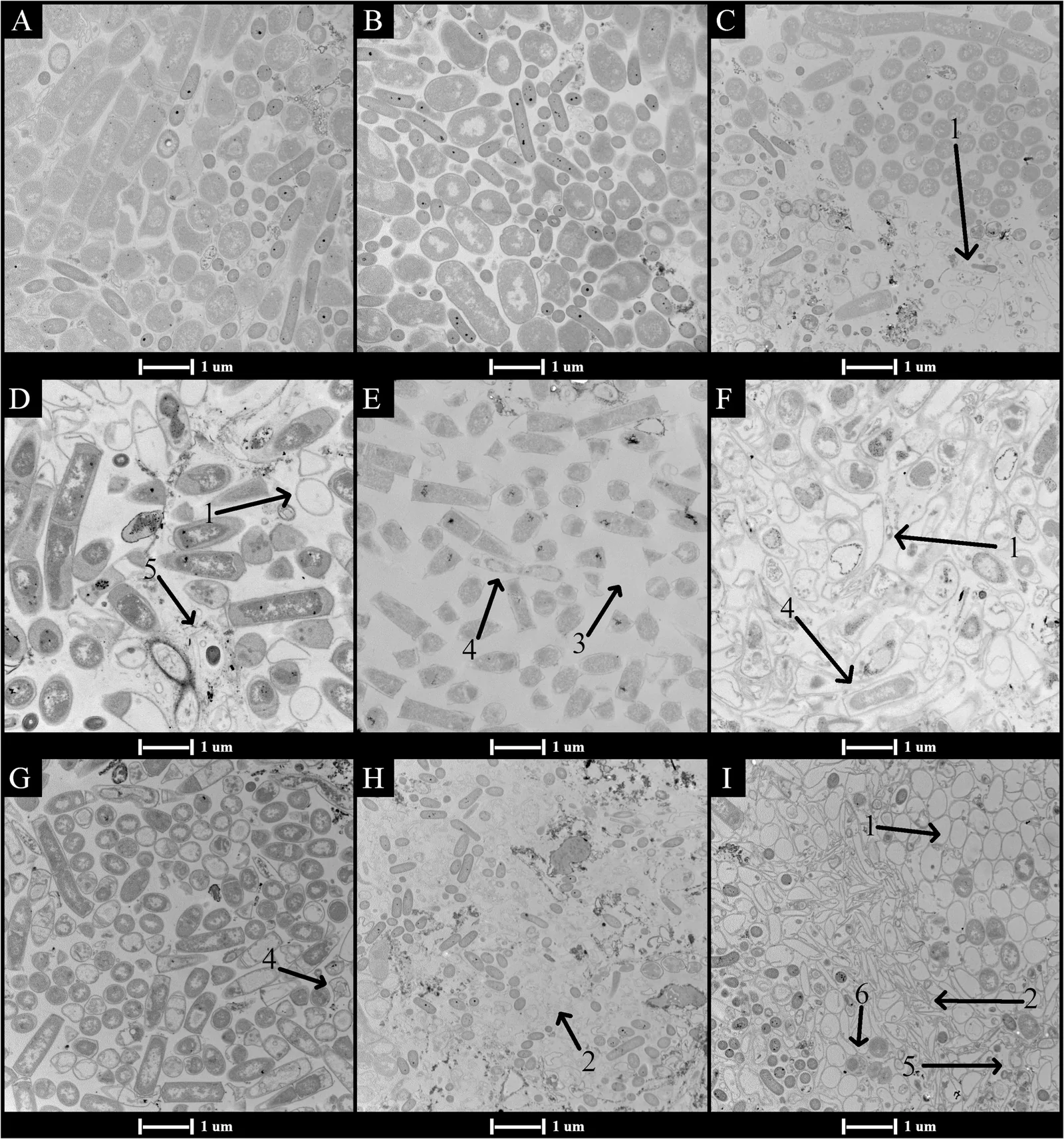
TEM pictures of AGS granules exposed to PFHxS and PFOA. **A**/**B**) CTRL+, granules that were fed with acetate for 5 days; **C**) CTRL-, granules that weren’t fed with acetate; granules that were exposed to **D**) 900 mg/L of PFHxS; **E**) 1,200 mg/L of PFHxS; **F**) 1,500 mg/L of PFHxS; **G**) 200 mg/L of PFOA; **H**) 300 mg/L of PFOA; **I**) 400 mg/L of PFOA. Numbered arrows point to: (1) hollowed cells husks; (2) dense debris areas; (3) wide gaps between cells; (4) plasmolysis; (5) cytoplasmic leakage; and (6) intact cells

Under control conditions, both negative (Fig. 2C) and positive (Fig. 2A/B), cells embedded in the granules showed a defined structure and morphology. The granule shows a patchwork of areas, with a variable uniformity in cell population. Negative controls though showed an increase in dead cells and debris compared to positive controls.

When granules were exposed to PFHxS at different concentrations (Fig. 2 from D to F), the microbes present show signs of increased cell death and structural damage. At 900 mg/L (Fig. 2D) the granular structure seems maintained, but cells show membrane disruption with loss of cytoplasmic material, with an overall increase in cell deformation. At 1,200 (Fig. 2E) and 1,500 (Fig. 2F) mg/L, the granule itself appears less dense in cells and connecting materials, and even cells that still appear alive show clear perturbation of their cell membrane and wall.

The phenomenon is similar with PFOA. From 200 to 400 mg/L (Fig. 2 from G to I) there is an increase in dead cells and morphological alterations, cells display classical membrane disruption and cytoplasmic outflow, particularly visible in Fig. 2H/I, yet the granule structure and density seem unchanged. Additional figures can be found in the Online Resources (Fig. S2 and S3).

### Effects of PFASs on Methane Producing Microbiome

#### Microbial Selection Under PFAS-related Exposure

**Fig. 3:**
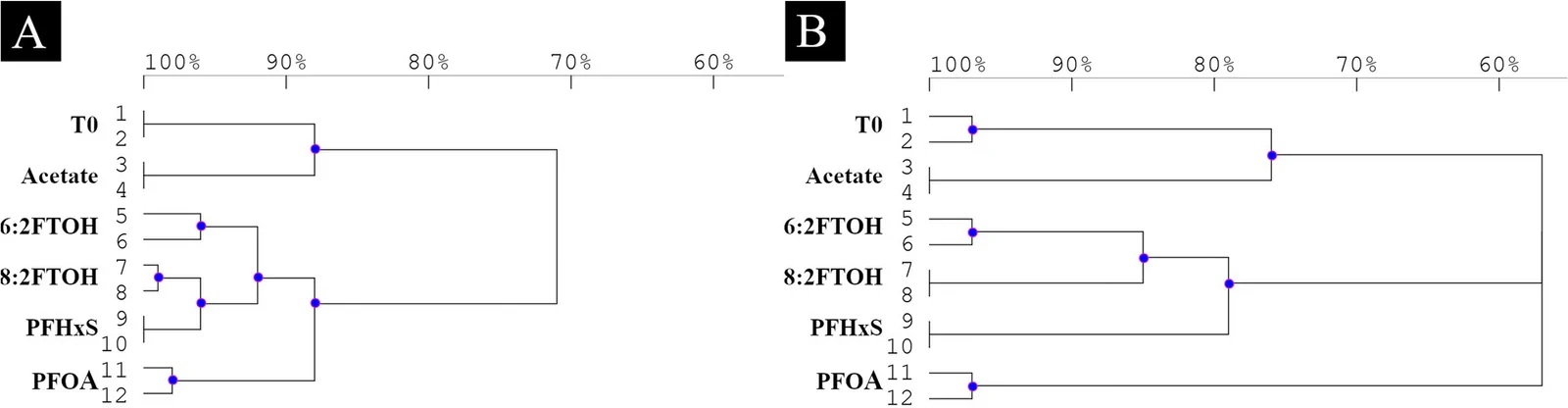
Dendrograms and homology associated from the DGGE bands pattern. (**A**) eubacterial and (**B**) archaeal population

DGGE was performed on a selection of samples to investigate the possible changes in microbial community composition after PFASs exposure (Fig. S4). All produced an elevated number of bands and the dendrograms built upon them show a similar pattern for both the archaeal and eubacterial communities (Fig. 3).

For the Archaea, the T0 and CTRL+ samples clustered together, sharing a similarity of 76% (Fig. 3A). Samples treated with the FTOHs and PFHxS share a higher similarity, 85% between the fluorotelomers and 79% with PFHxS. PFOA samples share a homology of only 57% with the others.

For the Eubacteria, T0 and CTRL+ clustered together with 88% similarity (Fig. 3B). The sample exposed to PFOA shows a higher affinity with the other treatments, sharing 88% similarity, even though they seem to cluster together with 96% similarity in the presence of PFHxS and 8:2FTOH treatments and 93% with 6:2FTOH.

#### Effects of PFASs Exposure on Microbial Diversity

The diversity of the microbial community changed in the AGS samples when exposed to PFASs (Table 1). Regarding the Archaea, all samples show an H index higher than the control except for the one exposed to PFHxS and reflect the results of the Chao1 index. The eubacterial community showed instead a higher change: all samples decreased in diversity as shown by the H index, except for the one exposed to PFHxS (Table 1). The evenness of the microbial population in the AGS samples was almost identical for the Archaea, while for the Eubacteria it had a higher discrepancy, with the AGS sample exposed to PFOA showing less diversity and homogeneity of species.

**Table 1:** Alpha diversity for the microbial community in PFASs exposed AGS measured through Chao1, Shannon diversity index (H) and Shannon equitability index (EH)

Community	Samples	Observed number of species	Chao1	H	E_H_
Archaea	CTRL+	167	167	4.93	0.029
	6:2FTOH	167	167	5.029	0.030
	8:2FTOH	170	170	5.134	0.030
	PFHxS	164	164	4.627	0.028
	PFOA	193	193	5.372	0.028
Eubacteria	CTRL+	175	175	6.055	0.035
	6:2FTOH	140	140	5.939	0.042
	8:2FTOH	168	168	5.642	0.034
	PFHxS	150	150	6.072	0.040
	PFOA	159	159	4.598	0.029

**Fig. 4:**
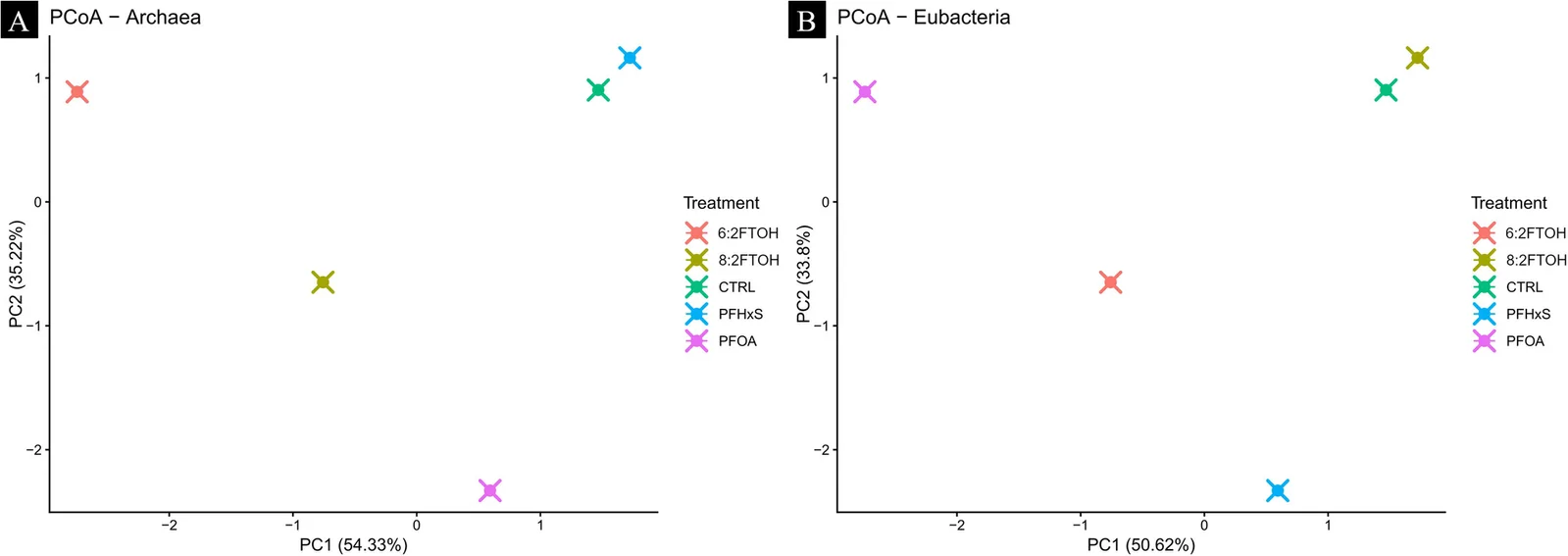
Principal Coordinates Analyses (PCoA) plots for (**A**) Archae and (**B**) Eubacteria, calculated on the weighted unifrac distance matrix (Beta diversity) presented in Table S2

Figure 4 reports the PCoA plots for the beta diversity calculated among the AGS samples for the archaeal and eubacterial communities (Table S2). PFHxS had the least effects in changing the archaeal composition (18.77% diversity), followed by 8:2FTOH, PFOA and 6:2FTOH. 8:2FTOH had the highest similarity with all conditions, being the closest to the control but also having between 27 and 30% of diversity with the other incubations. All conditions, except for 8:2FTOH, show a clear distinction with more than 40% of diversity with 6:2FTOH. Instead, for the Eubacteria, the control has the highest similarity with the PFHxS exposure, closely followed by 6:2FTOH, 8:2FTOH and lastly PFOA. In this case, PFOA exposure provides the highest diversity from all conditions, only 8:2FTOH showed a comparable effect, albeit smaller.

#### Changes in Archaeal Microbial Community

**Fig. 5:**
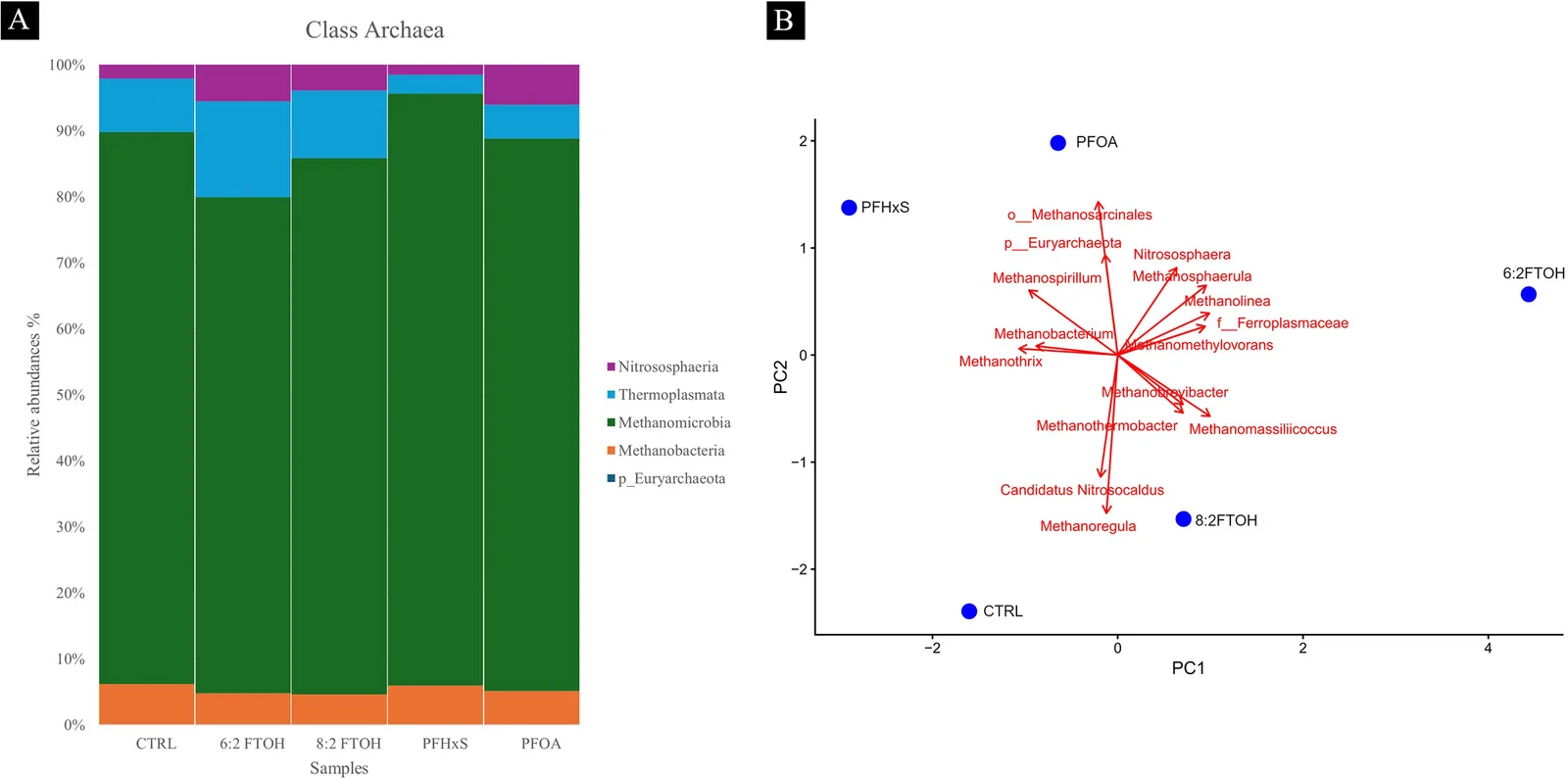
Relative abundances of the OTUs identified within the samples regarding the archaeal microbial community. (**A**) reports the results at a class identification level, while (**B**) the PCA plot at the genus level. When the identification wasn’t possible at the level required, results are preceded by a letter to indicate the most accurate identification level: p_ phylum, c_ class, o_ order and f_ family

The phylum of the *Euryarchaeota* dominated the Archaea community (Fig. 5), with the class of the *Metanomicrobia* being the most abundant (Fig. 5A). At the class level, and in terms of relative abundance, the control and the sample incubated in the presence of 8:2FTOH are the most similar. The *Euryarchaeota* for these two represent 96.7% and 94.6% of all the OTUs recovered, respectively, and are comprised of comparable amounts of the classes *Methanobacteria*, *Methanomicrobia* and *Thermoplasmata*. The sample treated with 2,000 mg/L of 6:2FTOH showed a decrease in the relative abundance of *Methanomicrobia* from 82.6% to 73.6% with respect to the control, and an increase in *Thermoplasmata* from 8.0% to 14.2% in relative abundance. An opposite trend is observed with 2,000 mg/L of PFHxS, where the relative abundance of *Methanomicrobia* increase to 88.3% and that of the *Thermoplasmata* decrease to 2.8%. The sample exposed to 600 mg/L of PFOA shows a slight decrease in *Methanomicrobia* to 78.6% and a further decrease of the relative abundance of *Thermoplasmata* to 4.8%.

Among the *Methanomicrobia*, the highest relative abundance is given by the genera *Methanolinea* and *Methanothrix*. The former shows an increase under all conditions tested. The control is composed of 16.7% of *Methanolinea*, a value that steadily increased under exposure to PFHxS, PFOA and 8:2FTOH up to 33.2% with exposure to 6:2FTOH. *Methanothrix* instead has similar relative abundances under control conditions and when exposed to PFHxS: 65.5% and 68% respectively, while decreasing with the other PFASs with the highest drop upon exposure to 6:2FTOH, reaching a relative abundance of 39.4%.

Aside, there are the *Thaumarchaeota* with the class of the *Nitrososphaeria* that was less represented in our samples and showed minimal variations upon PFAS exposure.

#### Changes in the Eubacterial Microbial Community

**Fig. 6:**
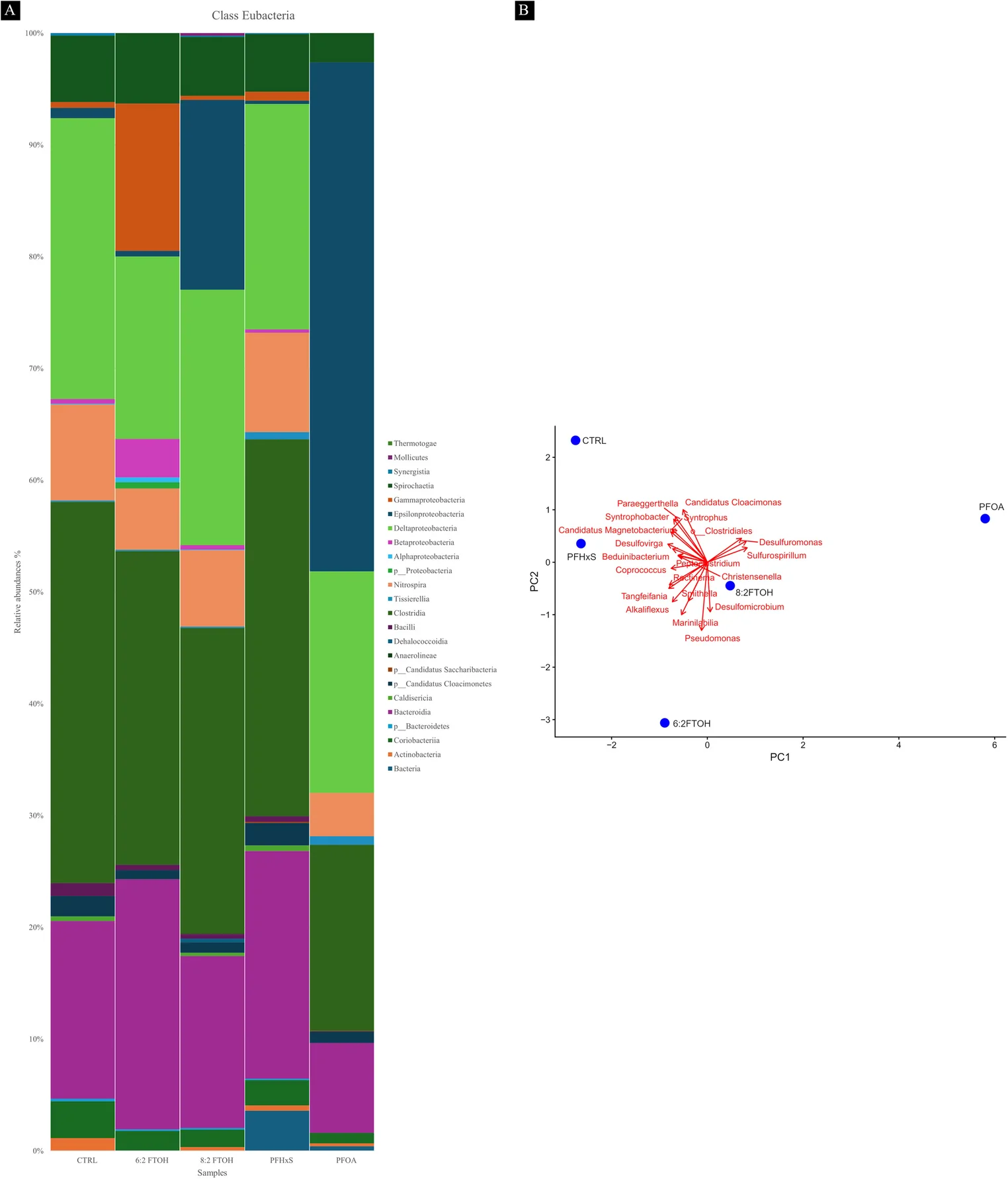
Relative abundances of the OTUs identified within the samples regarding the eubacterial microbial community. (**A**) reports the results at a class identification level, while (**B**) the PCA plot at the genus identification level. When the identification wasn’t possible at the level required, results are preceded by a letter to indicate the most accurate identification level: p_ phylum, c_ class, o_order and f_ family

In the eubacterial microbial community (Fig. 6), the most abundant classes are the *Bacteroidia* from the *Bacteroidetes* phylum, *Clostridia* from the *Firmicutes*, the *Delta*, *Epsilon* and *Gammaproteobacteria* from the *Proteobacteria* phylum and the *Spirochetia* from the *Spirochaetes* phylum (Fig. 6A). The *Bacteroidia* have similar proportions in both the control and the sample exposed to 8:2FTOH: 15.9% and 15.4% respectively. This value increases to 22.4% and 20.4% upon exposure to 6:2FTOH and PFHxS and decreases to 8.0% with PFOA. Among them, the higher proportion is given by the *Marinilabiliales* order, with the families of the *Marinilabiliaceae* and *Prolixibacteraceae*. The *Clostridia* instead decreased in relative abundance in every sample analysed, from 34.1% in the control to 33.7% with PFHxS, 28.1% with 6:2FTOH, 27.4% with 8:2FTOH and lastly 16.6% with PFOA. Among the *Proteobacteria*, the *Deltaproteobacteria* showed a decrease in relative abundance under all conditions tested. The control had a relative abundance of 25.1%, that lowers to 22.9% with 8:2FTOH, 20.2% with PFHxS, 19.8% with PFOA and 16.3% with 6:2FTOH. The orders of the *Desulfuromonadales* and the *Syntrophobacterales* have the highest representation for this class.

The *Epsilonproteobacteria* showed the highest variation: the control and the samples exposed to 6:2FTOH and PFHxS all have values lower than 1%, while their relative abundance increased in the samples exposed to 8:2FTOH and PFOA, respectively, to 17% and 45.5%. Of this class, the highest representation is from *Sulfurospirillum*, from the *Campylobacterales* order. For the *Gammaproteobacteria* the variation is mostly focused on 6:2FTOH, that expressed a relative abundance value of 13.2% almost all attributable to the *Pseudomonadaceae* with the genus *Pseudomonas*, while for all the others it was less than 1%. Lastly, the *Spirochaetia* were well represented among all samples investigated, with an average value of 5–6% relative abundance, that dropped to 2.6% when exposed to PFOA.

## Discussion

### Inhibition of Acetoclastic Methane Production by PFASs

Methane production is a process driven by Archaea and can be affected by a wide range of pollutants, including metals and microplastics, [28, 32–33]. In this study, both PFHxS and PFOA exerted a clear inhibitory effect to acetoclastic methanogenesis, although relatively high concentrations were required to reach significant inhibition (Table S1). The lower IC_50_ observed for PFOA (261 ± 33.5 mg/L) compared to PFHxS (1,091.9 ± 26.78 mg/L) indicates a higher toxicity of perfluorinated carboxylic acids relative to sulfonates under the tested conditions. Accordingly, the pH shift is also remarkably less pronounced when concentrations of PFOA or PFHxS rise above these thresholds (Table S1), reflecting reduced metabolic activity likely associated with cell damage or death [25].

Only a limited number of studies have investigated the effects of PFAS on the methane yields of AGS (Table S3)[11–13, 34–41]. For instance, Silva et al. (2022) reported a decrease in methane production of approximately 15% at 100 mg/l of PFOA [11]. Similarly, Jiao et al. (2022) suggested that inhibition of methanogenesis may arise from alterations in the expression of key enzymes and increased production of reactive oxygen species (ROS), with PFOA negatively affecting key enzymes like acetate kinase (AK), butyrate kinase (BK) and coenzyme F_420_ [34]. In addition, the amphiphilic and surfactant-like properties of PFAAs may promote interactions with cellular membranes [9, 10], increasing membrane permeability and destabilizing cellular structures, thereby contributing to the observed inhibition [34, 45].

Despite the lack of data at higher PFASs concentrations, the trend observed at 100 mg/L of PFOA is consistent with an initial inhibition followed by recovery. In our study, methane yield decreased during the first 24 h after exposure to 100 mg/L of PFOA, but recovered after 48 h of incubation. A similar recovery pattern was reported by Choi & Kan (2024) [40], suggesting that microbial communities may partially adapt to PFAS-induced stress over time. In contrast, FTOHs showed only marginal effects, even at concentrations as high as 2,000 mg/L. This behavior is consistent with their distinct physicochemical properties compared to fully fluorinated compounds. FTOHs are neutral and partially fluorinated molecules, generally exhibiting lower direct toxicity compared to fully fluorinated perfluoroalkyl acids, whose amphiphilic and ionized nature promotes stronger interactions with cellular membranes [9, 10]. Previous studies have shown that FTOHs can be transformed under methanogenic conditions, suggesting that their effects may be partly mediated by transformation products rather than the parent compounds, although this process is relatively slow and leads primarily to intermediate polyfluorinated compounds rather than terminal perfluorinated acids [39, 42]. Given the relatively short experimental duration (96 h), it is highly unlikely that fluorinated transformation products significantly contributed to the observed effects, although their role cannot be entirely excluded. Moreover, the compact structure of anaerobic granules may impose mass transfer limitations, further reducing both the bioavailability and transformation of these compounds [43, 44].

Although studies on PFHxS or FTOHs in AGS are still scarce, it is generally recognised that perfluorinated compounds are more toxic than polyfluorinated precursors. In this context, Choi & Kan [40] reported a higher toxicity of PFOA to PFOS at the same concentrations. The different impact of pfas on methanogenic activity can be attributed to their distinct physicochemical properties. fully fluorinated compounds such as pfoa and pfhxs are known to exhibit a strong surfactant behaviour due to their amphiphilic and ionized nature, enabling interactions with cell membranes and potentially disrupting their integrity [9, 10, 45]. In contrast, ftohs are neutral, partially fluorinated compounds [7, 8], which may result in reduced direct interactions with cellular membranes and a lower immediate impact on microbial activity. our results are in agreement with these findings, showing that FTOHs exert only minor inhibitory effect, while PFOA displays a stronger inhibitory potential than PFHxS under comparable conditions.

### Alteration of Granular Structure and Microbial Morphology

The TEM observations (Fig. 2) revealed that PFASs exposure not only interferes with metabolic activity, but also alter cellular morphology and granule structure. Both PFOA and PFHxS induced plasmolysis and cell wall alterations, while PFHxS in particular appeared to increase the gaps between cells in the matrix, suggesting a distruption of structural cohesion (Fig. 2D). These alterations may impair mass transfer processes and microbial interactions within the granule, further contributing to the observed functional inhibition.

Across the tested concentration range (200–600 mg/L for PFOA and 900–1500 mg/L for PFHxS), the effects seemed to be similar, but the magnitude was enhanced with the increase in concentration. At the lowerst concentrations, of PFASs abnormal cell morphological alterations appearedwere rare and sparse and localized, whereas higher concentrations resulted in more widespread afflicted areas widened by increasing the concentration of PFASs. Still, even at the highest concentrations, cells and areas of the granule that seemed undamaged regions of intact cells were still present, indicating a certain degree of heterogeneity in the response of the microbial community (Fig. 2).

The observed cellular damage is consistent with previous findings. For example, Liu et al. (2016) reported plasmolysis and cytoplasmic leakage in *Escherichia coli* when exposed to 500 mg/L of PFHxS or 15 mg/L of PFOA. This could be explained by PFOA being able to disrupt the balance of the antioxidant system, increasing the damage caused by ROS leading to cell inactivation and death through apoptosis [34] and their detergent-like properties, enhancing membrane fluidity [45].

### PFAS Induced Shifts in the Microbial Community

The DGGE analyses indicated that PFASs exposure induces shifts in microbial community composition, in agreement with previous studies on PFOA [40, 45–47]. These changes suggest that PFAS not only directly inhibit methanogenic activity, but also indirectly affect process performance through microbial selection and community restructuring.

The microbial community showed a clear distinction between different samples. The Archaea community was dominated by the genera *Methanothrix* and *Methanolinea*, and the relative abundance of both changed when exposed to PFASs. The relative abundance of *Methanolinea* increased under all conditions tested, similarly to what Cao et al. (2022) reported, but the higher variation was with the FTOHs (Fig. 4). In contrast, the relative abundance of *Methanothrix* decreased in most conditions, except under PFHxS exposure, and especially so with 6:2FTOH. This genus is particularly important given its role in providing mechanical strength to the granular structure [48], and its decline may therefore be directly linked to the observed reduction in methane production as well as the integrity of the granules.

In the bacterial community, PFASs exposure generally led to an increase in relative abundance, except in PFHxS incubations. Notably, PFOA exposure resulted in a marked increase in *Proteobacteria* relative abundance (+ 276%), largely driven by the genus *Sulfurospirillum*. This genus is associated with nitrate reduction and was recently linked with the biodegradation of PFOS and chlorinated solvents by Lorah et al. (2024), and it was also found before to increase in abundance in AGS exposed to perchlorate [48–50]. Its enrichment may indicate a shift toward alternative metabolic pathways under PFAS stress, potentially at the expense of methanogenic activity. Similarly, the genus *Pseudomonas* increased substantially from being virtually absent in all samples to represent a relative abundance of 10.4% in the presence of 2,000 mg/L of 6:2FTOH. This genus is well known for its metabolic versatility and has been frequently associated with the biotransformation of fluorinated compounds, including fluorotelomers [51]. Its enrichment may therefore reflect an adaptive response to PFAS exposure.

Interestingly, Archaea and Eubacteria communities exhibited opposite trends in alpha diversity. PFHxS exposure reduced archaeal diversity, while other PFASs slightly increased it. Conversely, eubacterial diversity decreased in most conditions, except in PFHxS-treated samples, where it remained comparable to the control (Table 1). Shifts in alpha diversity have been linked to ecosystem stability, implying that changes may impact redundant processes and the ability of an ecosystem, in our case the AGS, to face environmental changes and disturbances [52]. Thus, even though the microbial community composition only partially aligns with the one in this study, there is consensus that PFASs decrease the microbial diversity of exposed samples [12, 34, 35].

Beyond their direct impact on methanogenic activity, PFAS exposure appears to influence other key microbial processes involved in biogeochemical cycling. Several of the taxa affected in this study are not exclusively linked to methanogenesis, but play important roles in carbon, nitrogen, and sulfur transformations [22] and may have broader implications for the degradation of co-occurring contaminants. The observed decrease in *Clostridia* and *Deltaproteobacteria*, which are involved in primary fermentation and syntrophic metabolism, suggests a disruption of upstream processes that provide substrates for methanogenesis [20, 21]. In particular, members of the orders *Syntrophobacterales* and *Desulfuromonadales* are known to participate in syntrophic interactions and sulfur-related metabolic processes, and their decline may reflect a weakening of cooperative metabolic networks within the granules. Notably, the marked decrease in *Syntrophobacter* across all PFAS treatments suggests a disruption of syntrophic fatty acid oxidation, a process tightly coupled to methanogenesis [21]. This reduction may contribute to the observed inhibition of methane production. Similarly, the decline of fermentative taxa affiliated with *Prolixibacteraceae* (like the genus *Tangfeifania*) and *Christensenellaceae* (like the genus *Beduinibacterium*) indicates a potential impairment of primary fermentation processes, further constraining substrate supply to methanogens [20]. Conversely, the marked increase in *Epsilonproteobacteria*, particularly the genus *Sulfurospirillum*, indicates a shift toward alternative metabolic pathways. This genus has been associated with the reduction of sulfur and nitrogen compounds and has been previously linked to PFAS-related processes and with the degradation of chlorinated compounds [49]. Similarly, the increase in *Pseudomonas*, especially in FTOH-treated samples, may be associated with its ability to metabolize fluorinated compounds and participate in xenobiotic degradation pathways [51].

At the archaeal level, the increase in hydrogenotrophic methanogens (like *Methanolinea*) alongside the decline of acetoclastic taxa (as *Methanothrix*) suggests a functional shift in methane production pathways, likely reflecting altered substrate availability and system stress [16–18]. The consistent decrease in *Candidatus Nitrosocaldus*, together with the differential response of *Nitrososphaera*, suggests that ammonia-oxidizing archaea may exhibit variable sensitivity to PFAS, potentially leading to shifts in nitrification pathways [52]. In addition, the reduction of *Candidatus Magnetobacterium* may reflect alterations in microbial populations associated with redox-stratified environments, as members of this group are related to magnetotactic bacteria involved in iron biomineralization processes [53, 54].

Recent studies suggest that bacterial assemblages may be more sensitive to PFAS-driven perturbations, showing pronounced shifts in community composition and the enrichment of stress-tolerant and metabolically versatile taxa. In contrast, archaeal communities tend to exhibit lower taxonomic variability but pronounced functional shifts, particularly in the balance between acetoclastic and hydrogenotrophic methanogenesis [55, 56]. The differential response observed between archaea and eubacteria may be explained by their distinct ecological roles, physiological sensitivity, and spatial organization within the granules. Methane production by Archaea depends on a narrow range of substrates and syntrophic interactions [57]. Consequently, PFAS-induced disturbances mat result in functional reconfiguration, as observed by the shift from acetoclastic (*Methanothrix*) to hydrogenotrophic (*Methanolinea*) methanogens (Fig. 4). In contrast, bacterial communities exhibit higher metabolic versatility and include taxa involved in multiple cycles and degradation processes. This functional diversity may strengthen community-level restructuring under PFAS stress, leading to the selection of more tolerant or opportunistic taxa such as *Pseudomonas* and *Sulfurospirillum*, while sensitive fermentative groups such as *Clostridia* decline. Additionally, the spatial organization within the granules may contribute to these differences, as bacteria are more exposed to external stressors through the EPS matrix, whereas archaea are often located in the more protected inner regions [55, 56].

Altogether, these results indicate that PFAS impact extends beyond methanogenesis, affecting multiple functional guilds and potentially reshaping carbon, nitrogen, and electron transfer pathways within anaerobic granular sludge, in agreement with previous observations of PFAS-induced microbial community restructuring [12, 22, 40, 47].

## Conclusions

Different PFAS exert distinct effects on AGS based on concentration and exposure time. Among the compounds tested, PFHxS and PFOA were able to completely inhibit the production of methane from acetate, with PFOA being the most asserting. In contrast, FTOHs exhibited only minor short-term impacts on acetoclastic methanogenic activity, although slight inhibition was observed. The effects of PFASs on AGS extend beyond methane inhibition and involve multiple levels of biological organization. TEM observations showed that increasing concentrations of PFHxS and PFOA compromise cell integrity through plasmolysis, leading to damaged cells and increased intercellular spacing within the granule matrix. These structural alterations may impair microbial interactions and mass transfer processes within the granules. PFAS exposure also induced significant shifts in microbial community composition, with more pronounced changes observed in the bacterial fraction. In particular, the enrichment of genera such as *Pseudomonas* and *Sulfurospirillum* suggest the selection of stress-tolerant populations and alternative metabolic pathways under PFASs stress. Overall, these findings indicate that PFAS not only inhibit methanogenic activity, but also reshape microbial communities and disrupt key metabolic interactions within anaerobic systems. Such multilevel effects may have broader implications for anaerobic carbon cycling and the stability of microbial ecosystems. Notably, the high concentrations required to induce significant inhibition suggest that anaerobic granular sludge exhibits a considerable degree of resilience to PFAS exposure. However, the observed structural and functional alterations highlight the potential for sublethal effects that may influence long-term system performance and ecosystem functioning.

## Supplementary information

Below is the link to the electronic supplementary material.

Supplementary PDF 1 (PDF 3.03 MB)

## Data Availability

The datasets generated and analysed during the current study are available in the NCBI repository, [https://www.ncbi.nlm.nih.gov/bioproject/PRJNA1416243/]
