# Supplementary material for: Effects of Per- and Polyfluoroalkyl Compounds (PFASs) on Anaerobic Granular Sludge: Methane Production and Microbial Community Composition
Source: Microb Ecol. 2026 Jul 9;89:182. doi: 10.1007/s00248-026-02793-4 (PMC13631125; doi:10.1007/s00248-026-02793-4)
Supplement: Supplementary file 1 [file 248_2026_2793_MOESM1_ESM.pdf]

***Table S1**: Effects of PFASs on methane production by AGS and the pH of their medium upon completion of the experiment. The pH of the buffer at the start of the test period was 7.28 (± 0.05). CTRL+ stands for the positive controls. Values of methane yields that may have resulted inferior to the negative controls were reported as 0.*

**Conditions**			**Methane yields (%)**			
	[PFAS] mg/L	pH (96 h)	24 h	48 h	72 h	96 h
**CTRL+**	0	7.60 ± 0.08	100 ± 12.4	100 ± 4.7	100 ± 15.08	100 ± 13.47
**6:2FTOH**	100	7.54 ± 0.06	100.94 ± 6.86	107.87 ± 22.78	87.86 ± 11.82	87.86 ± 8
	600	7.48 ± 0.02	101.17 ± 1.98	108.69 ± 1.47	88.53 ± 1.19	88.53 ± 1.19
	2,000	7.54 ± 0.06	91.08 ± 13.52	98.83 ± 9.12	80.5 ± 7.43	80.5 ± 7.43
**8:2FTOH**	100	7.5 ± 0.01	90.6 ± 0.93	96.18 ± 7.24	108.26 ± 10.84	101.34 ± 5.78
	600	7.54 ± 0.11	104.91 ± 2.35	105.89 ± 6.36	132.05 ± 6.18	102.9 ± 4.13
	2,000	7.48 ± 0.02	71.89 ± 1.16	103.51 ± 1.5	115.54 ± 0.82	105.99 ± 1.13
**PFHxS**	100	7.48 ± 0.04	104.96 ± 11.55	109.56 ± 14.2	125.05 ± 7.24	112.11 ± 9.64
	600	7.51 ± 0.09	95.53 ± 6.05	118.39 ± 2.84	132.57 ± 1.91	111.9 ± 2.63
	900	7.51 ± 0.02	20.1 ± 2.54	97.53 ± 15.05	115.8 ± 7.7	125.79 ± 6.43
	1,200	7.44 ± 0.03	4.06 ± 0.55	6.48 ± 1.22	16.06 ± 4.54	23.07 ± 0.69
	1,500	7.42 ± 0.03	2.8 ± 1.45	2.25 ± 2.01	4.54 ± 4.2	6.14 ± 3.77
	2,000	7.29 ± 0	0	0	0	0
**PFOA**	100	7.52 ± 0.01	89.13 ± 1.48	99.02 ± 0.14	115.56 ± 1.95	105.93 ± 1.53
	200	7.55 ± 0.15	75.1 ± 9.92	98.27 ± 1.64	96.24 ± 2.7	93.77 ± 1.35
	250	7.49 ± 0.01	9.14 ± 0.66	91.06 ± 2.38	98.65 ± 6.82	102.42 ± 4.32
	300	7.39 ± 0.08	6.91 ± 4.74	10.65 ± 6.12	12.27 ± 9.64	23.59 ± 18.54
	350	7.32 ± 0.03	4.02 ± 8.54	13.38 ± 31.53	0	0
	400	7.29 ± 0.02	2.2 ± 12.07	0	12.58 ± 21.86	0
	600	7.24 ± 0.06	0	0	0	0

Table S2: *Weighted unifrac distances matrix (Beta diversity) calculated for the microbial community among the AGS samples exposed to different PFASs.*

Community	Samples	CTRL	6:2FTOH	8:2FTOH	PFHxS	PFOA
**Archaea**	CTRL+	0	0.4463	0.1877	0.1024	0.3064
	6:2FTOH	0.4463	0	0.2838	0.4871	0.4353
	8:2FTOH	0.1877	0.2838	0	0.2725	0.3051
	PFHxS	0.1024	0.4871	0.2725	0	0.3272
	PFOA	0.3064	0.4353	0.3051	0.3272	0
**Eubacteria**	CTRL+	0	0.1881	0.2299	0.1078	0.5851
	6:2FTOH	0.1881	0	0.3364	0.1975	0.6206
	8:2FTOH	0.2299	0.3364	0	0.2473	0.3812
	PFHxS	0.1078	0.1975	0.2473	0	0.6078
	PFOA	0.5851	0.6206	0.3812	0.6078	0

Table S3: Effects of PFASs on various types of anaerobic and aerobic sludges.

System	Exposure	PFAS concentration	Main effects	Reference
**Anaerobic granular sludge**	PFOA	0.1 µg/L – 10 mg/L	Increase in accumulated methane yields. Decrease in hydrolysis rate. PFOA adsorption. Inhibition of methanogenic bacteria.	[13]
	PFOA	20 – 100 mg/L	Inhibit COD removal rate. Increase in EPS abundance.	[12]
	PFOA<br>PFOS	0.1 – 100 mg/L,<br>0.1 – 80 mg/L	Decrease in methane yields.	[11]
	6:2FTOH<br>8:2FTOH<br>PFHxS<br>PFOA	100 – 2000 mg/L	Decrease in methane yields. Microbial cell death and granules structural variation. Microbial composition shift.	This study
**Anaerobic digestion system**	PFOA<br>PFOS	0.1 – 100 mg/L	Decrease in methane yields.	[40]
**Waste activated sludge**	PFOA	8.5 – 170 mg/kg TS	Decrease in methane yields and enzymes activity. Increase in ROS production.	[34]
**Aerobic granular sludge**	PFOS	0.1 – 1 mg/L	Decrease in denitrification and phosphorous uptake and release. Increase in competition between microorganisms. Decrease in structural stability. EPS increased abundance.	[35]
	PFOS	0.1 – 0.5 – 5 mg/L	PFOS adsorption. Improved denitrification.	[36]
	PFOA	0.05 – 0.5 – 2 mg/L	Decrease removal of nitrogen and phosphorous. EPS increased abundance.	[37]
	PFPeA<br>PFOA<br>PFBS<br>PFDS	3 µg/L	PFAS adsorption.	[38]

	PFOA	0.1 mg/L	EPS increased abundance.<br>Decrease in denitrification.	[39]
	PFOA	0.1 – 0.5 – 1 mg/L	Decrease in nitrogen and phosphorous removal.	[41]

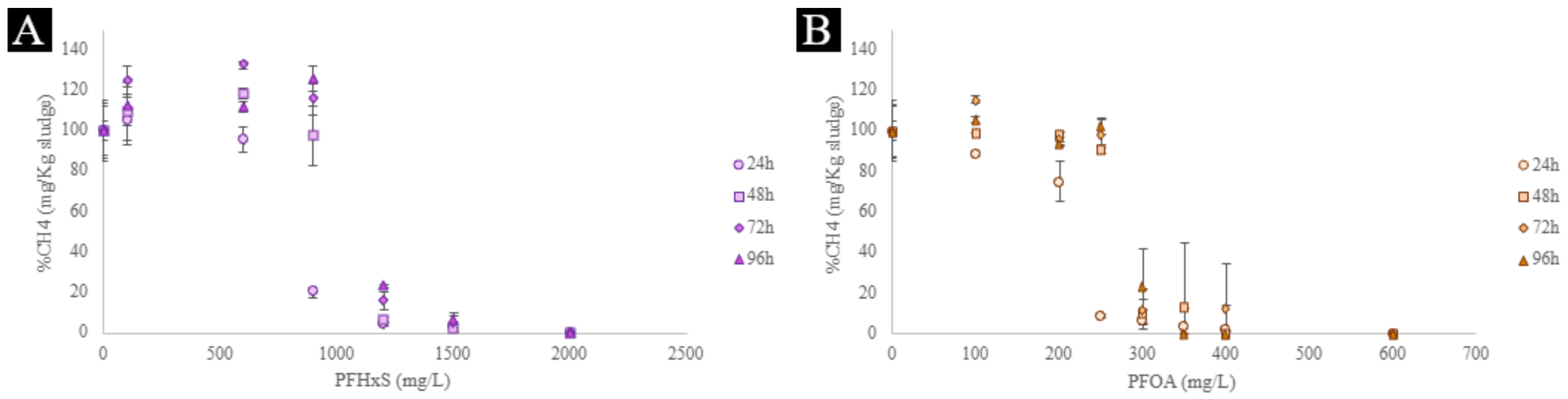

Fig. S1*: Methane yields, normalized on the control conditions, collected from tests conducted with PFHxS and PFOA. Here, 0 and 100% are respectively the amount of methane observed under negative and positive control conditions, i.e. without or with acetate as the substrate. Results for A) PFHxS and B) PFOA are reported for the time of sampling.*

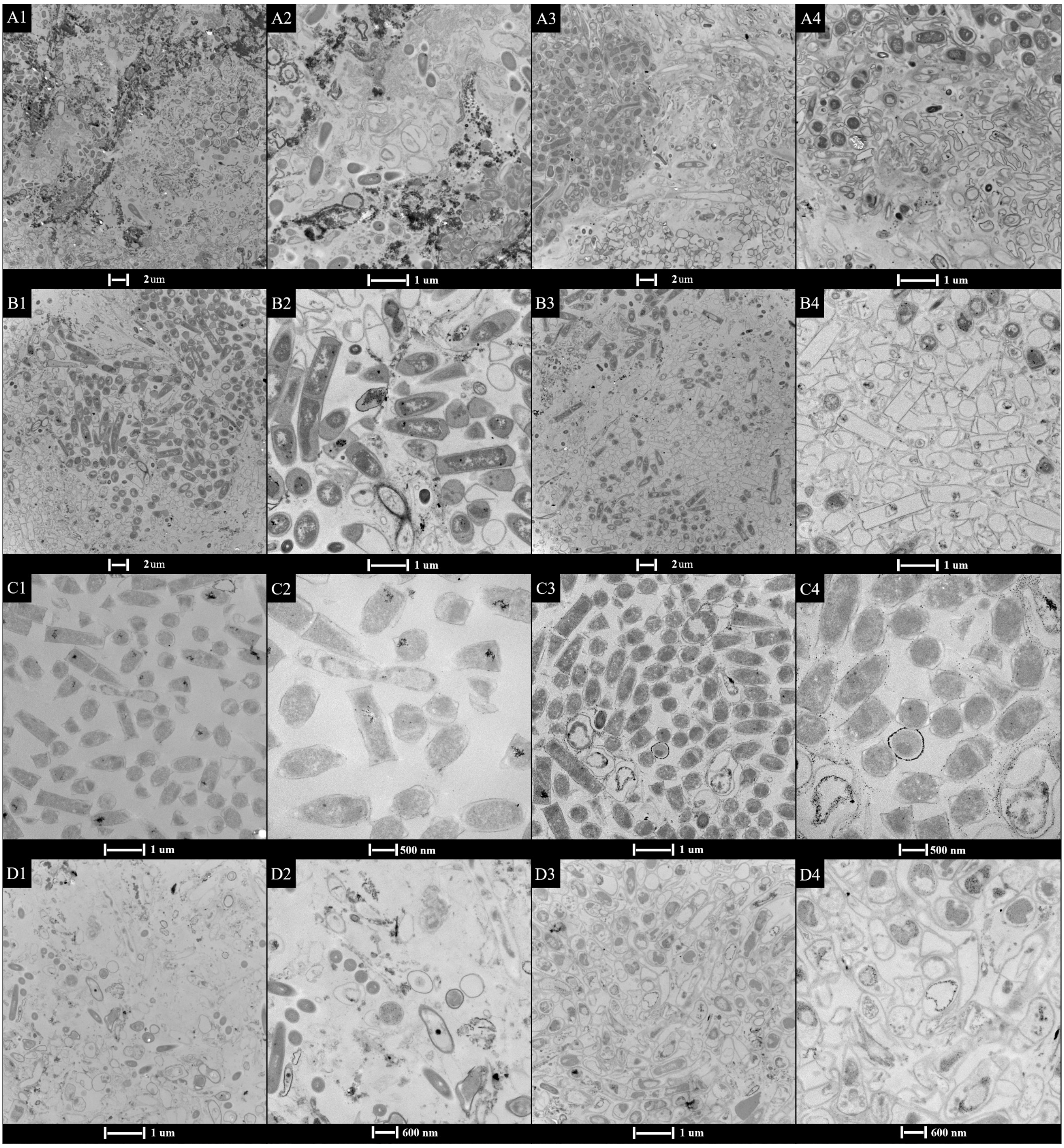

Fig. S2: *TEM pictures of granules subjected to different PFHxS concentrations. A1-A2) CTRL +, granules that were fed with acetate for 5 days; A3-A4) CTRL -, granules that weren't fed with acetate; B) second row of pictures, granules that were exposed to 900 mg/L of PFHxS; C) third row of pictures, granules that were exposed to 1200 mg/L of PFHxS; D) fourth row of pictures, granules that were exposed to 1500 mg/L of PFHxS. Under all conditions, except for CTRL-, the AGS was fed with acetate at 30 mM.*

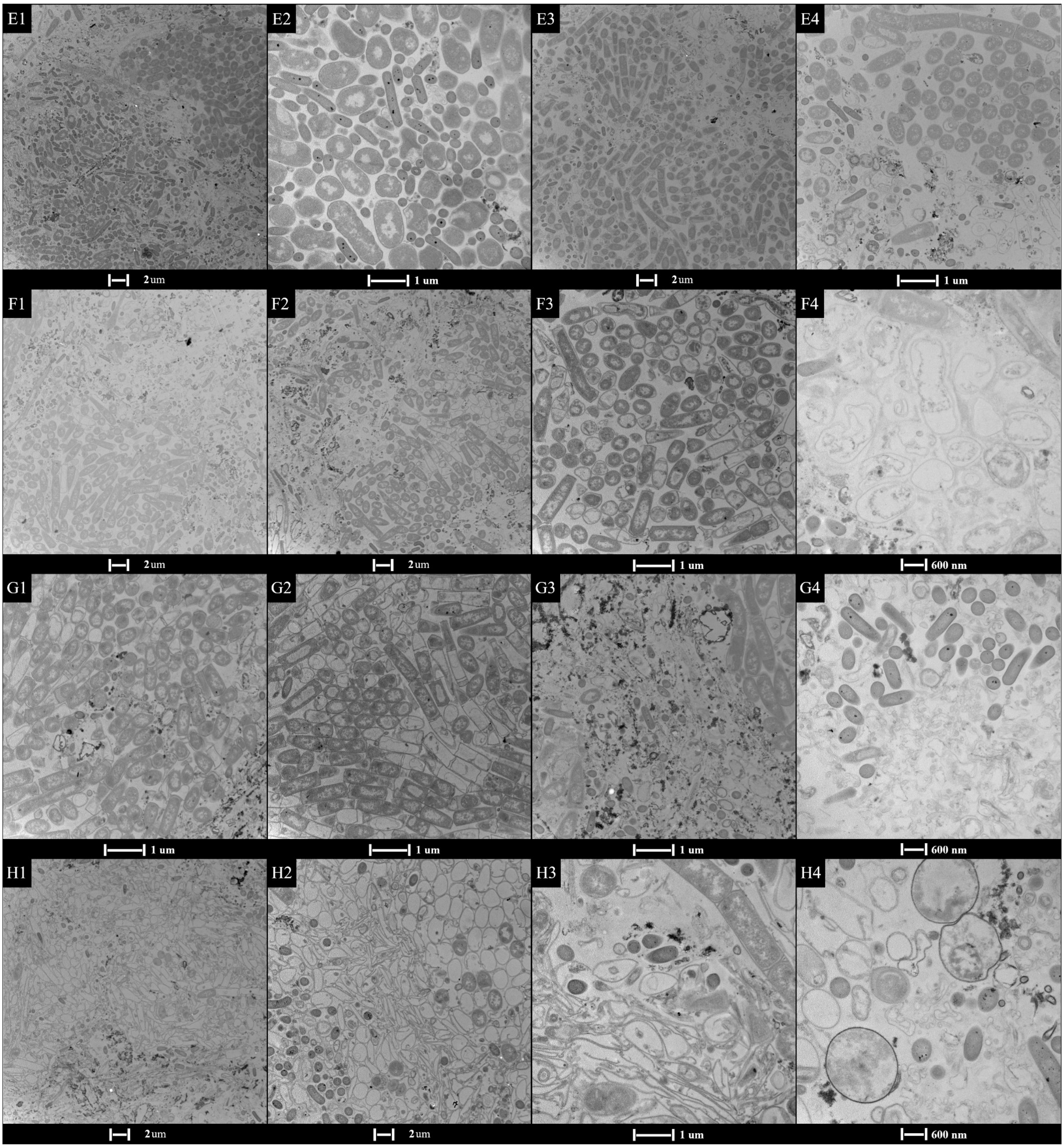

Fig. S3*: TEM pictures of granules subjected to different PFOA concentrations. E1-E2) CTRL +, granules that were fed with acetate for 5 days; E3-E4) CTRL -, granules that weren't fed with acetate; F) second row of pictures, granules that were exposed to 200 mg/L of PFOA; G) third row of pictures, granules that were exposed to 300 mg/L of PFOA; H) fourth row of pictures, granules that were exposed to 400 mg/L of PFOA. Under all conditions, except for CTRL-, the AGS was fed with acetate at 30 mM.*

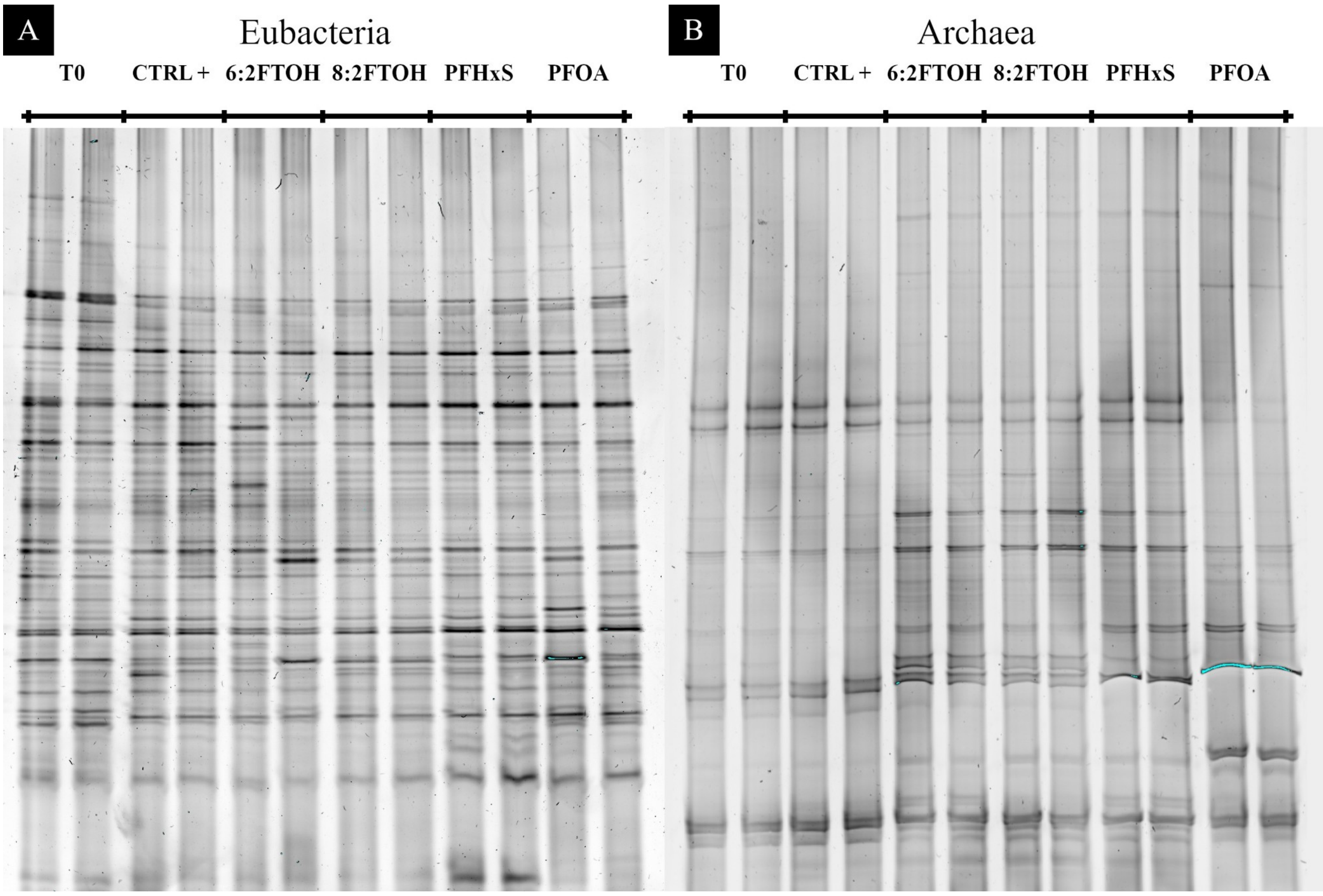

Fig. S4: *DGGE bands obtained for the A) Eubacteria, and B) Archaea.*
